# Gastrointestinal tuberculosis: clinical image

**DOI:** 10.11604/pamj.2023.45.60.39839

**Published:** 2023-05-25

**Authors:** Vikas Raghuvanshi, Vishal Balchand Padwale

**Affiliations:** 1 Department of Medicine, Jawaharlal Nehru Medical College, Datta Meghe Institute of Higher Education and Research, Sawangi (Meghe), Wardha, Maharashtra, India,; 2 Department of Gastroenterology, Jawaharlal Nehru Medical College, Datta Meghe Institute of Higher Education and Research, Sawangi (Meghe), Wardha, Maharashtra, India

**Keywords:** Gastrointestinal tuberculosis, interferon-gamma release assay, colonoscopy

## Image in medicine

A 65-year-old male came to our hospital complaining of abdominal pain in the right lumbar region for 2 months with lethargy. He had history of loss of appetite for one month with no history of fever, diabetes, tuberculosis, weight loss, or any surgeries. He has been a chronic alcoholic for the last 30 years. He had a history of dark-colored stools for a week. The patient did not receive BCG vaccination at his birth. Pallor was present in the palpebral conjunctiva, cardiovascular and respiratory system has no abnormality. On abdominal examination, mild tenderness in the right lumbar region otherwise the abdomen was soft on palpation with no organomegaly. His haemoglobin level was 8%, peripheral smear showed normocytic, normochromic anemia with few microcytes, and stool examination revealed occult blood. HIV and HBsAg tests were negative. Colonoscopy showed a circumferential ulcerated, narrowed ileocecal valve with a pulled-up caecum suggestive of abdominal tuberculosis. Multiple biopsies were taken to rule out dysplasia. The anal canal, rectum, sigmoid colon, descending colon, transverse colon, and ascending colon were visualized and their mucosa appeared normal. Computed tomography of abdomen showed mesenteric lymph node enlargement suggestive of intestinal tuberculosis. Interferon-gamma release assay, polymerase chain reaction, and Mantoux test were positive suggestive of tuberculosis, and fecal calprotectin level was normal. Chest X-ray revealed no abnormalities. The patient started with antitubercular therapy (ATT) according to national protocol i.e. rifampicin, isoniazid, pyrazinamide, and ethambutol were prescribed for four months, followed by rifampicin and isoniazid for two months along with vitamin B6.

**Figure 1:**
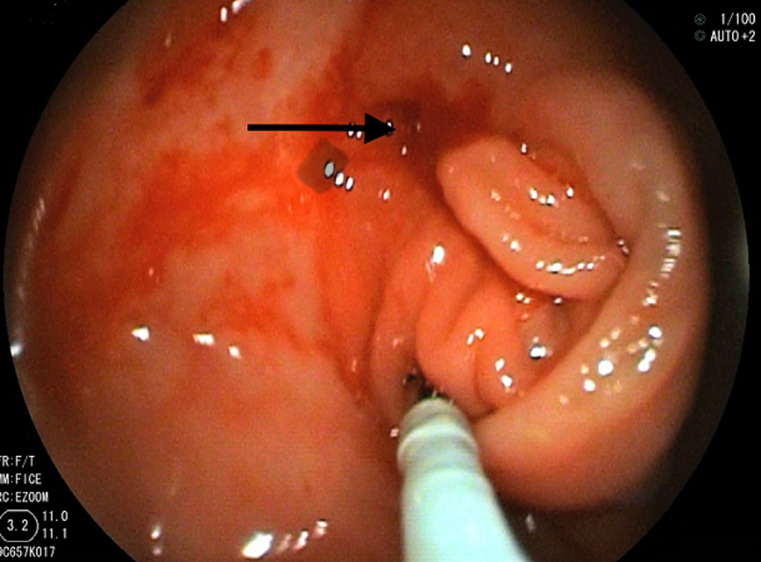
colonoscopy showing a circumferential ulcerating, narrowing ileocecal valve with a pulled-up caecum suggestive of abdominal tuberculosis

